# Supplementary material for: Behavior-dependent directional tuning in the human visual-navigation network
Source: Nat Commun. 2020 Jun 26;11:3247. doi: 10.1038/s41467-020-17000-2 (PMC7320013; doi:10.1038/s41467-020-17000-2)
Supplement: Supplementary file 1 — Supplementary Information [file 41467_2020_17000_MOESM1_ESM.pdf]

Supplementary material:

Behavior-dependent directional tuning in the human visual-navigation network

Authors: Matthias Nau, Tobias Navarro Schröder, Markus Frey, Christian F. Doeller

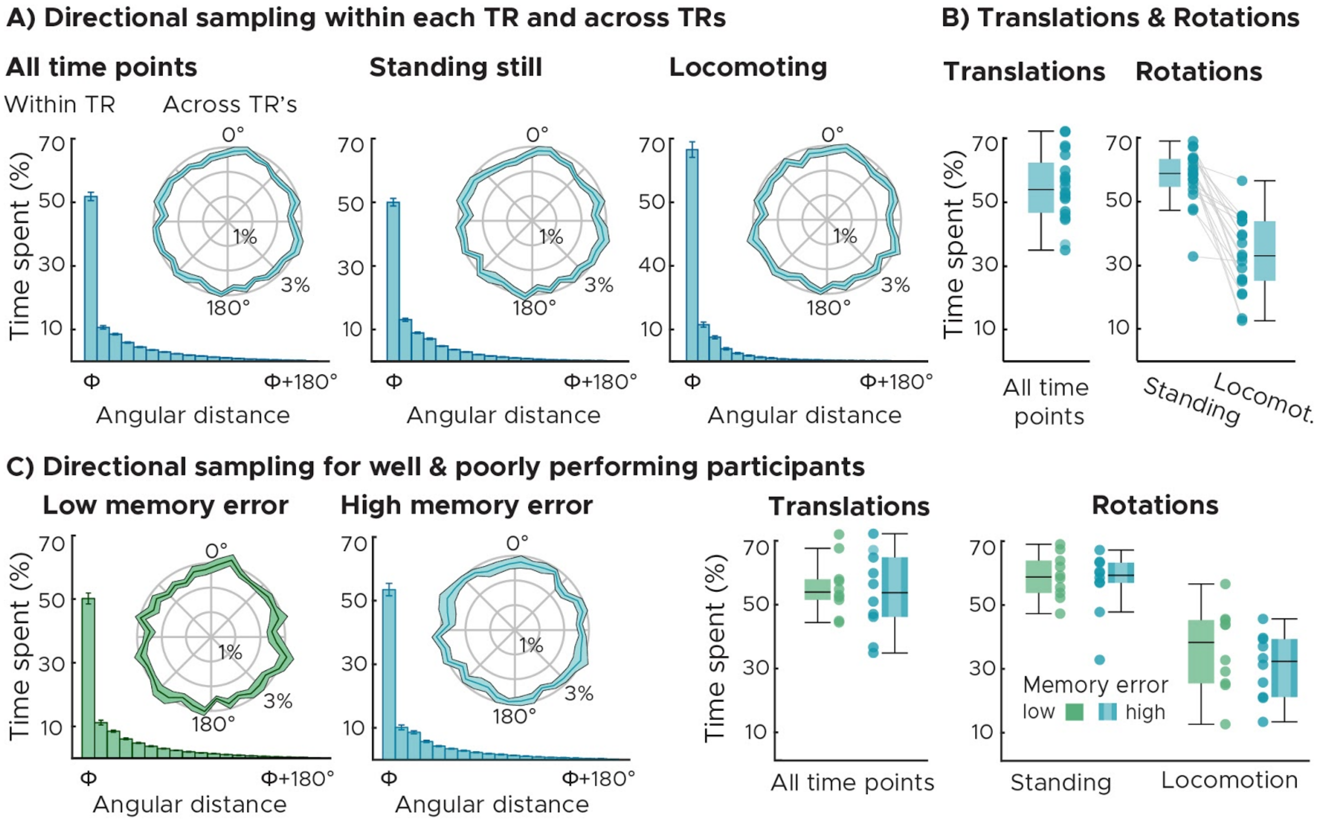

Supplementary figure 1. Navigation behavior (n = 20). A) Directional sampling across all time points (left), stationary periods only (middle) and locomotion periods only (right). Directional sampling expresses the time spent facing into each direction in percent of total experiment time. The bar plots depict the across-participant mean & SEM within each fMRI acquisition (TR). Data were pooled according to the angular distance to the predominant direction (Φ, first bar). Within each TR, participants' faced into one predominant direction for around 50% of the total experimental time. The time spent facing into the predominant direction differed between stationary and locomotion periods (two-tailed permutation-based paired ttest results: t(19) = 6.12, p = 0.0001, k = 10000). Polar plots depict the directional sampling across the entire experiment (line: mean, shaded area: SEM). Across the experiment, sampling was matched across directions, showing that there were no asymmetric spatial cues that biased navigation behavior (rmANOVA results for all time points: F(35, 665) = 0.77 , p = 0.834, while standing still: F(35, 665) = 0.87, p = 0.681 and while locomoting: F(35, 665) = 0.79, p = 0.806). B) Translations and rotations. Participants locomoted around 50% of the total experimental time (left panel). While locomoting, participants spent less time rotating than while standing still (right panel) (two-tailed permutation-based paired ttest results: t(38) = 7.95, p = 0.0001, k = 10000). We plot single-participant data and group-level whisker-and-box plots (center, median; box, 25th to 75th percentiles; whiskers, 1.5 × interquartile range, n = 20 participants). C) Directional sampling across participant groups. There were no differences in directional sampling in neither of the two participant groups across TRs (rmANOVA results: low-memory-error group: F(35, 315) = 0.87, p = 0.675, high-memory-error group: F(35, 315) = 0.55, p = 0.984). The two participant groups spent equal amount of time locomoting (t(18) = 0.41, p = 0.690), and rotating (while locomoting: t(18) = 0.55, p = 0.625 and while standing still: t(18) = 1.00, p = 0.338) and facing towards one predominant direction within each TR (t(18) = -1.26, p = 0.224, k = 10000). All p-values reported here are uncorrected unless stated otherwise. We plot single-participant data and group-level whisker-and-box plots (center, median; box, 25th to 75th percentiles; whiskers, 1.5 × interquartile range, n = 2 x 10 participants). Source data are provided as a Source Data file.

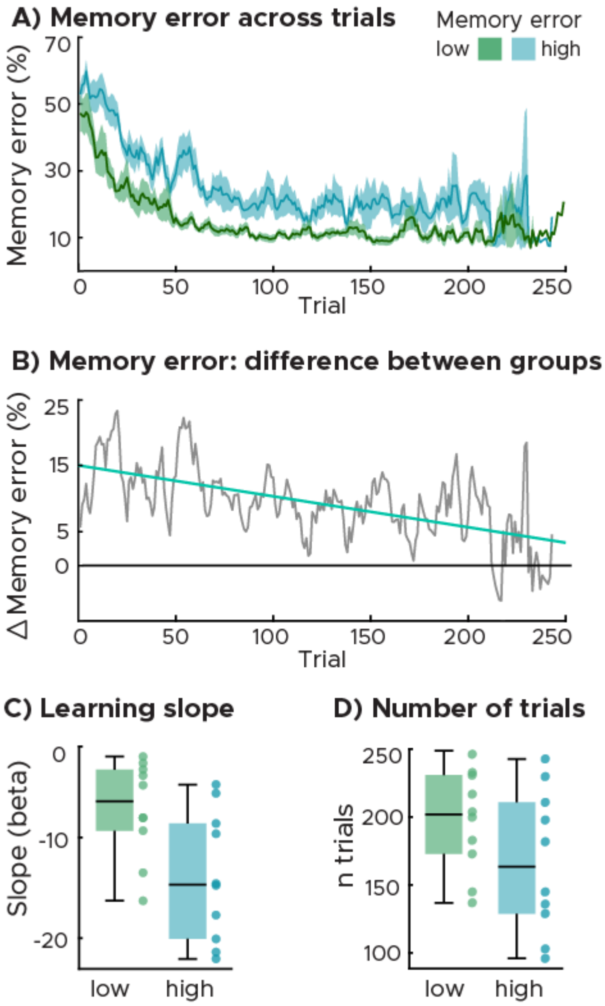

Supplementary figure 2: A) Memory error across trials for the two participant groups (n = 2 x 10 participants) differing in the median memory error. The memory error is the Euclidean distance between true and remembered object location expressed in virtual vertices. Data were smoothed using a running average kernel of 5 trials. B) Differences in memory error between groups (n = 2 x 10 participants). Zero constitutes no difference. Both groups converge on the same level of performance in the course of the experiment. C) Learning slopes. Regression slopes of a linear line fitted to the raw memory error scores across trials of each participant. Participants with higher median memory error showed steeper slopes (two-tailed permutation-based unpaired ttest results: $t(18) = 2.65$, $p = 0.019$, uncorrected), indicating that the memory error improved faster than in the low-memory-error group. D) Number of trials. Participants with low memory error performed slightly more trials than the ones with high memory errors, the difference between groups was however small ($t(18) = 1.46$, $p = 0.137$, uncorrected). C,D) We plot single-participant data and group-level whisker-and-box plots (center, median; box, 25th to 75th percentiles; whiskers, 1.5 × interquartile range, n = 2 x 10 participants). Source data are provided as a Source Data file.

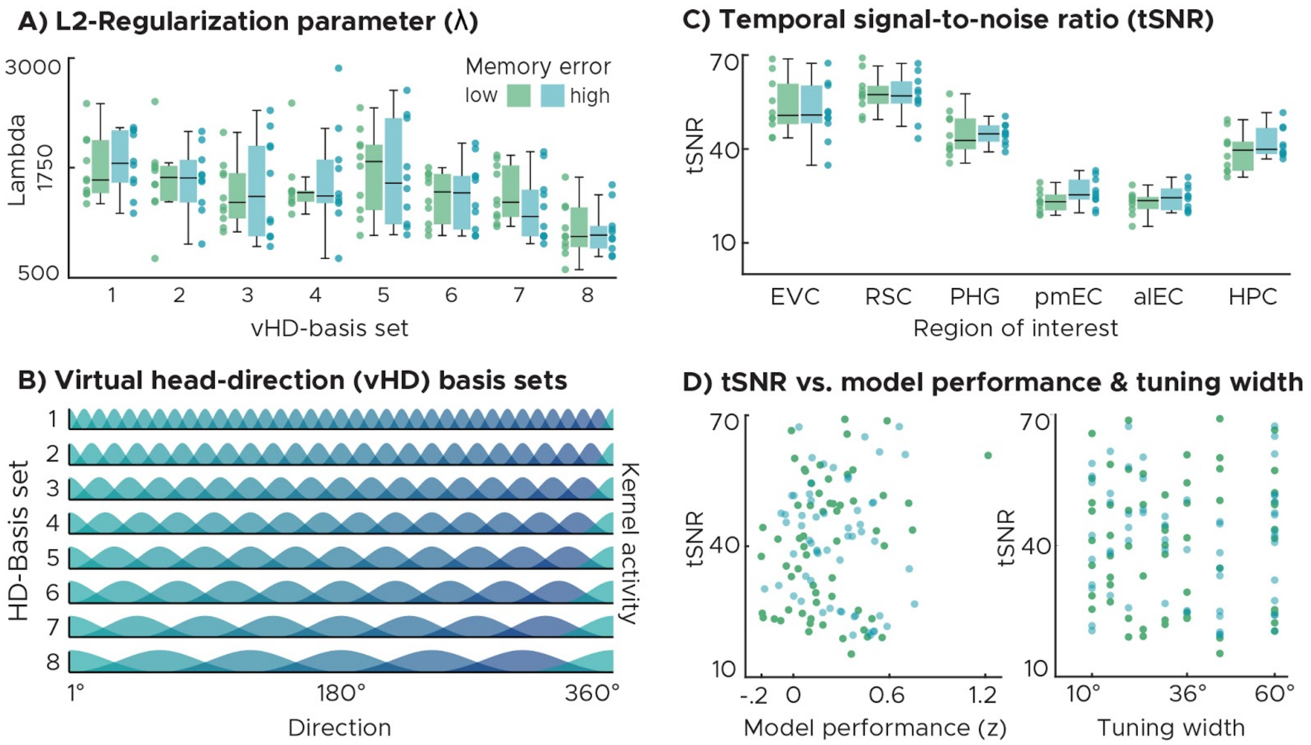

Supplementary figure 3: Model parameters and data quality. A) L2-regularization parameters (λ) for all basis sets and the two participant groups (differing in memory error, n = 2 x 10 participants). Each dot represents λ of a single participant and basis set averaged across all ROIs (Fig. 4). Group-level whisker-boxplots of the same data were added (center, median; box, 25th to 75th percentiles; whiskers, 1.5 × interquartile range). Lambda depended on the basis set (rmANOVA results: $F(7, 126) = 11.80$, $p = 1.7 \times 10^{-11}$) as expected, but not on participant group ($F(1, 18) = 0.0037$, $p = 0.952$). There was no interaction between the two ($F(7,126) = 0.37$, $p = 0.920$). B) Histogram depiction of all basis sets used. Each Gaussian represents one directional kernel covering the full 360° with 1° resolution. Basis sets differed in kernel width and spacing. Resulting regressors were scaled between 0 and 1. C) Temporal signal-to-noise ratio (tSNR) across ROIs and participant groups (n = 2 x 10 participants). We plot group-level whisker-boxplots (center, median; box, 25th to 75th percentiles; whiskers, 1.5 × interquartile range) and single participant data of the average tSNR of each region. There were differences in tSNR across ROIs (rmANOVA results: $F(5, 90) = 258.29$, $p = 9.1 \times 10^{-52}$), but not across participant groups ($F(1, 18) = 0.082$, $p = 0.777$) and there was no interaction between the two ($F(5, 90) = 1.34$, $p = 0.255$). D) Scatter plots for tSNR over model performance (left) and estimated tuning width (right). Dots represent the average tSNR of each region and participant. Neither model performance (Spearman correlation: rho = 0.076, p = 0.409), nor tuning width (rho = -0.059, p = 0.525) correlated with tSNR. All p-values reported here are uncorrected unless stated otherwise. Source data are provided as a Source Data file.

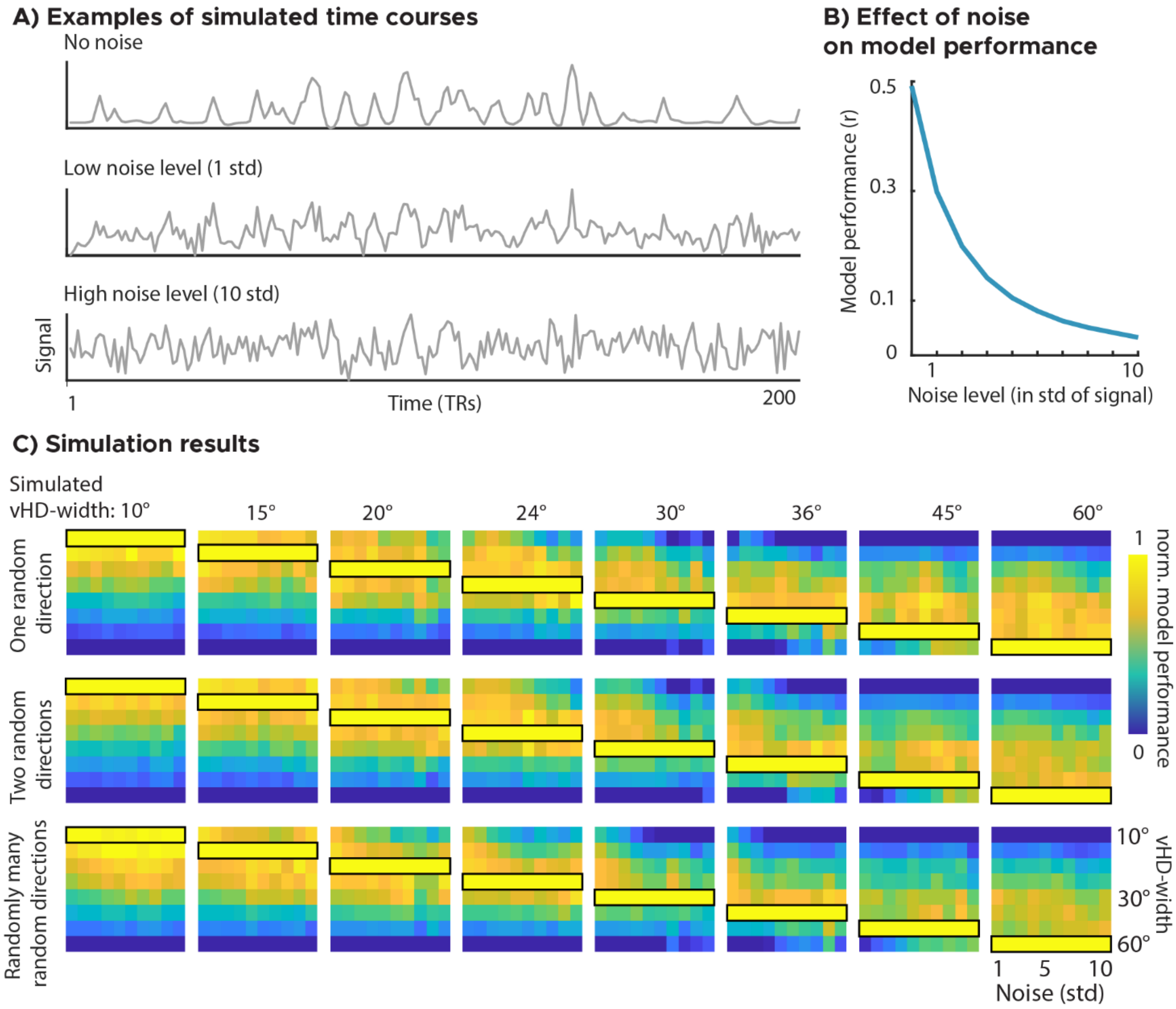

Supplementary figure 4: Virtual head direction (vHD) simulations. A) We simulated voxel time courses with known vHD-tuning properties by combining the vHD of a randomly chosen participant with simulated vHD-tuning profiles (unimodal, bimodal, random directional tuning). Plotted here are time courses of a bimodally tuned voxel (60°) at three noise levels (0, 1 and 10 standard deviations of the time course). We modeled 2500 voxels with 5 scanning runs for each tuning profile, noise level and tuning width combination. B) Effect of noise. We depict the model performance over noise levels averaged across 2500 bimodally tuned voxels and tuning widths (blue line, SEM across voxels hidden behind line). The model performed better at lower noise levels. C) Simulation results for all tuning profiles, tuning widths and noise levels. The black outline marks which tuning width was expected to show the highest model performance. If a tuning width of e.g. 10° was simulated, the kernel width of 10° should have led to the best model performance. This is the case for all tuning profiles, noise levels and tuning widths tested. The effect of noise was normalized for visualization. Source data are provided as a Source Data file.

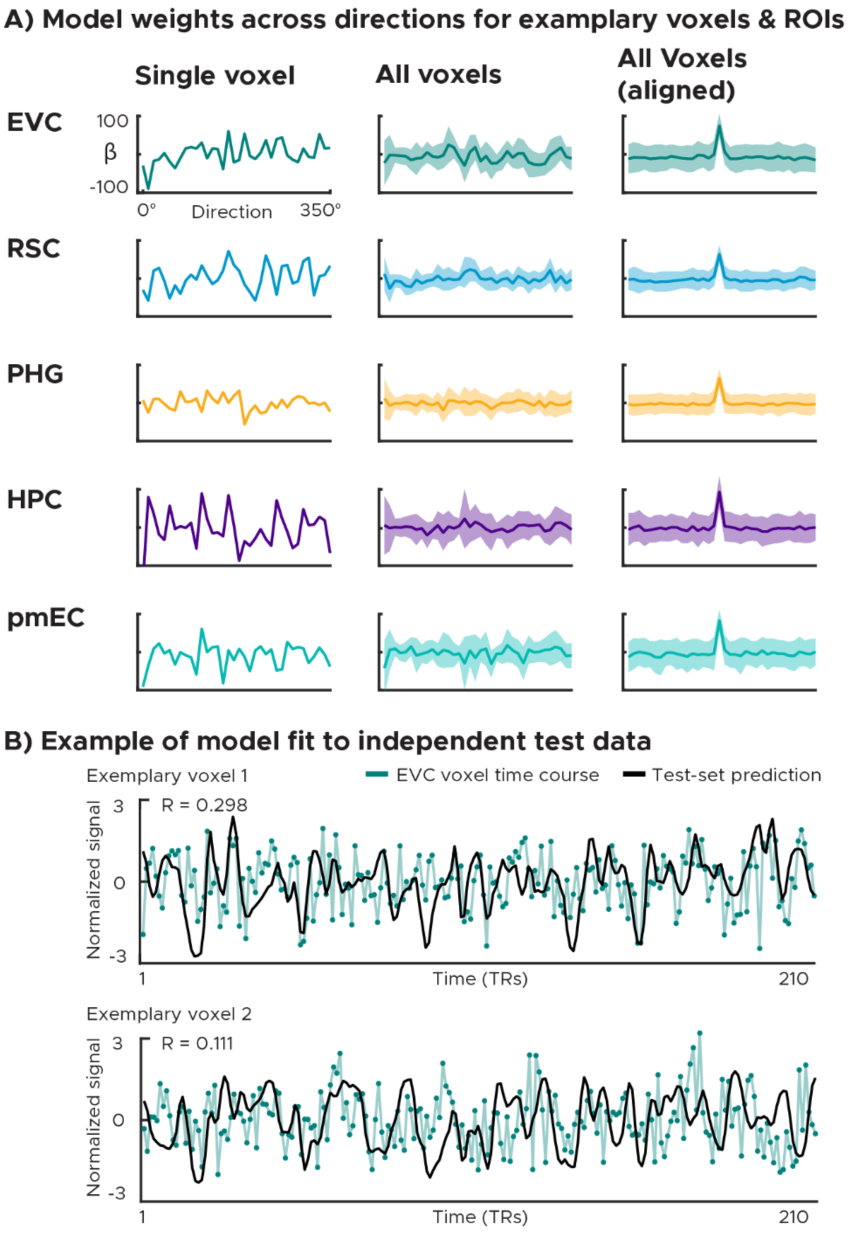

Supplementary figure 5: Tuning profiles and time course prediction for randomly selected sample voxels and regions of interest (ROIs). A) We plot the model weights across directions for one exemplary voxel of each ROI (left panel) and the model weights averaged across voxels of these ROIs (middle panel). To test whether there are tuning profiles that were consistent over voxels (e.g. uni-, bi-, trimodality) but averaged out across voxels, we additionally aligned the peak model weight across voxels (right panel). We plot the mean (solid line) and one standard deviation (shaded area) across voxels. ROIs: early visual cortex (EVC), retrosplenial cortex (RSC), parahippocampal gyrus (PHG), hippocampus (HPC) and posteromedial entorhinal cortex (pmEC). This figure shows that different voxels have distinct tuning profiles and prefer different directions. B) Example model fit to independent test data. We depict the predicted time course (black) of two sample voxels from the early visual cortex (EVC) superimposed onto the actually observed time course of these voxels in the test run. The model weights used to build the predicted time course were estimated on independent training data. All time courses were z-scored. Source data are provided as a Source Data file.

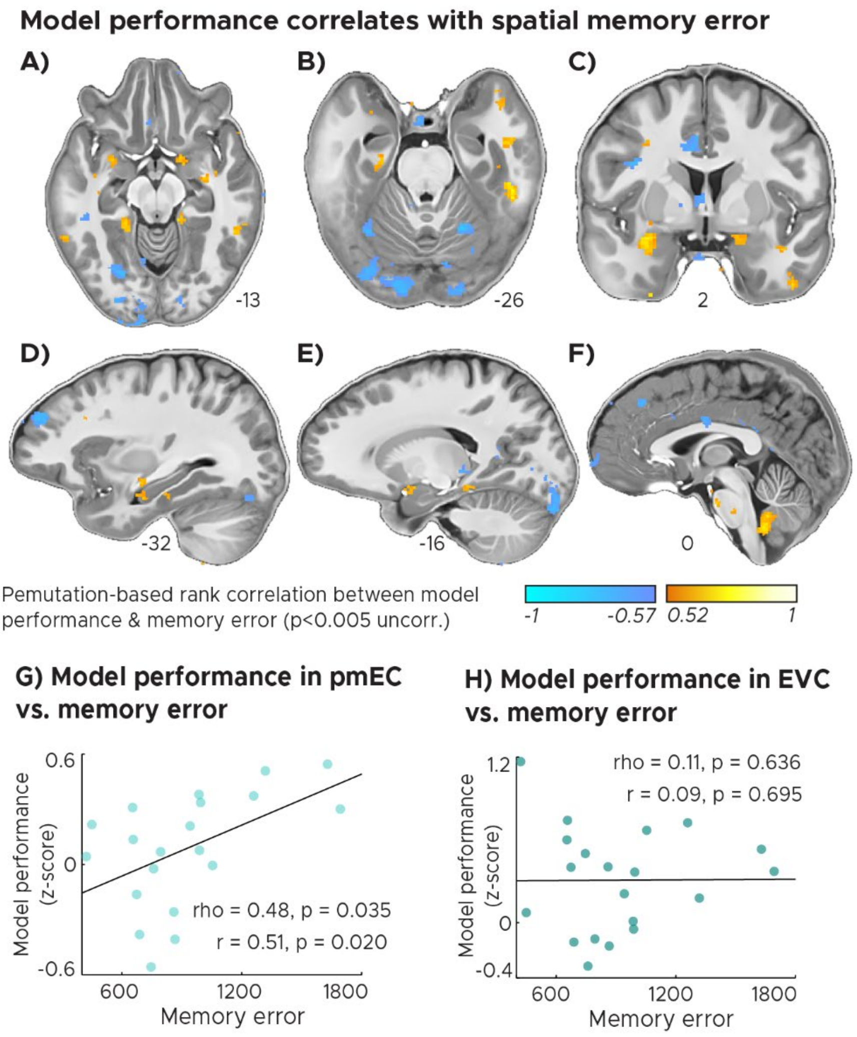

Supplementary figure 6: Across-participant permutation-based rank-correlation between the model performance (see Fig. 3) and across trial median memory error (Fig. 1). A-F) We plot Spearman's correlation coefficients (Rho) thresholded at p<0.005 uncorrected for visualization and overlaid on the group-average T1 template at T1 resolution. We observed a positive correlation between model performance and memory error in the parahippocampal gyrus (A,B,D,E) and anterior medial temporal lobe (A,B,C,D,E) including posterior entorhinal cortex (B) and anterior hippocampus (D), as well as in the ventral cerebellum (F). Negative correlations could be observed in the ventral occipital lobe (A,B,D,E), frontal lobe (D,F), the medial cingulate cortex (C) and the posterior thalamus (E). G-H) Scatter plot of posteromedial entorhinal cortex (pmEC) and early visual cortex (EVC) model performance at group-optimal tuning width of 45° over across-trial median memory error (Fig. 1). Least-square line as well as permutation-based rank & linear-correlation coefficients and uncorrected p-values were added. There is a correlation between model performance and memory error in pmEC, but not in the EVC. Source data are provided as a Source Data file.

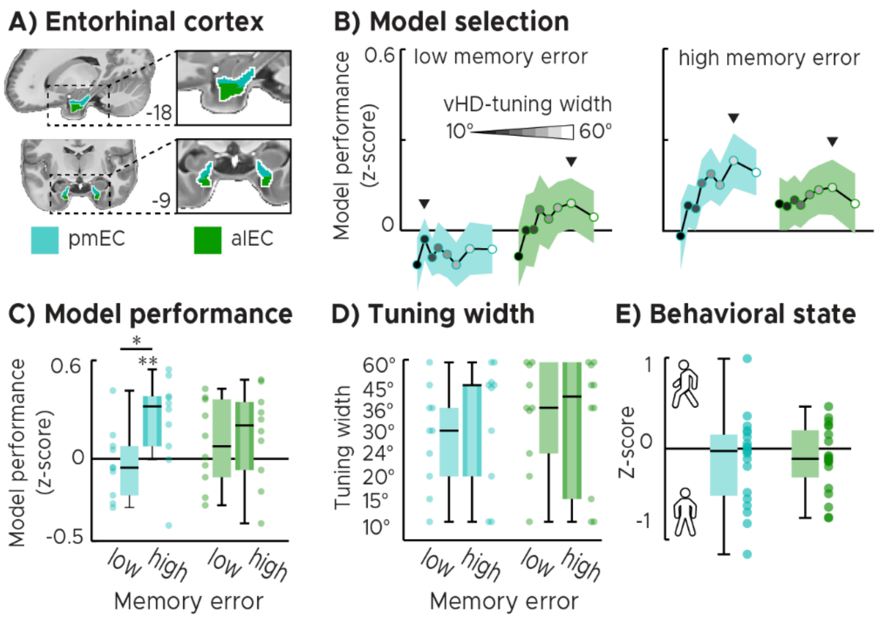

Supplementary figure 7: Comparison between entorhinal cortex subdivisions. A) Regions of interests: posteromedial (pmEC) vs. anterolateral entorhinal cortex (alEC). B) Model selection: We plot the model performance (Z-score) for all basis sets and the two participant groups (n = 2 x 10 participants). The black line and the shaded area represent the mean and SEM across participants. Each dot represents the group-average model performance for one basis set, with darker colors representing narrow kernels and lighter colors representing wider kernels. The following kernel widths were tested: 10°, 15°, 20°, 24°, 30°, 36°, 45°, 60°. For each ROI on group-level, we selected the best performing basis set as the optimal model to be tested. Also see Fig. 5B. C) Optimal model performance selected in B for the two (high- and low-memory-error) participant groups. We plot single participant data and group-level whisker-boxplots (center, median; box, 25th to 75th percentiles; whiskers, 1.5 × interquartile range, n = 2 x 10 participants). We observed directional tuning in pmEC in participants with high memory error and a difference between groups (low memory error: $t(9) = -0.42$, $p = 0.661$; high memory error: $t(9) = 2.59$, $p = 0.020$, $p_{FDR} = 0.040$, $t(19) = 2.32$, $p = 0.036$). In alEC, neither directional tuning nor the difference between groups could be observed (Low : $t(9) = 1.09$, $p = 0.146$; high: $t(9) = 1.58$, $p = 0.075$; contrast: $t(19) = 0.44$, $p = 0.655$). Also see Fig. 5C. D) Optimal tuning width. Similar to B,C, we plot the tuning width that led to the highest memory performance selected on individual participant level (see Fig. 5D) for the two participant groups (n = 2 x 10 participants). E) Behavioral state analysis. Model performance during locomotion and stationary periods. Positive values indicate that voxel time courses in the ROI could be better predicted when participants locomoted. We plot the difference in model performance as single participant data and group-level whisker-boxplots (center, median; box, 25th to 75th percentiles; whiskers, 1.5 × interquartile range, n = 20 participants). There is no effect of locomotion per se on the tuning in pmEC (and alEC which was not directionally tuned in our task). Source data are provided as a Source Data file.

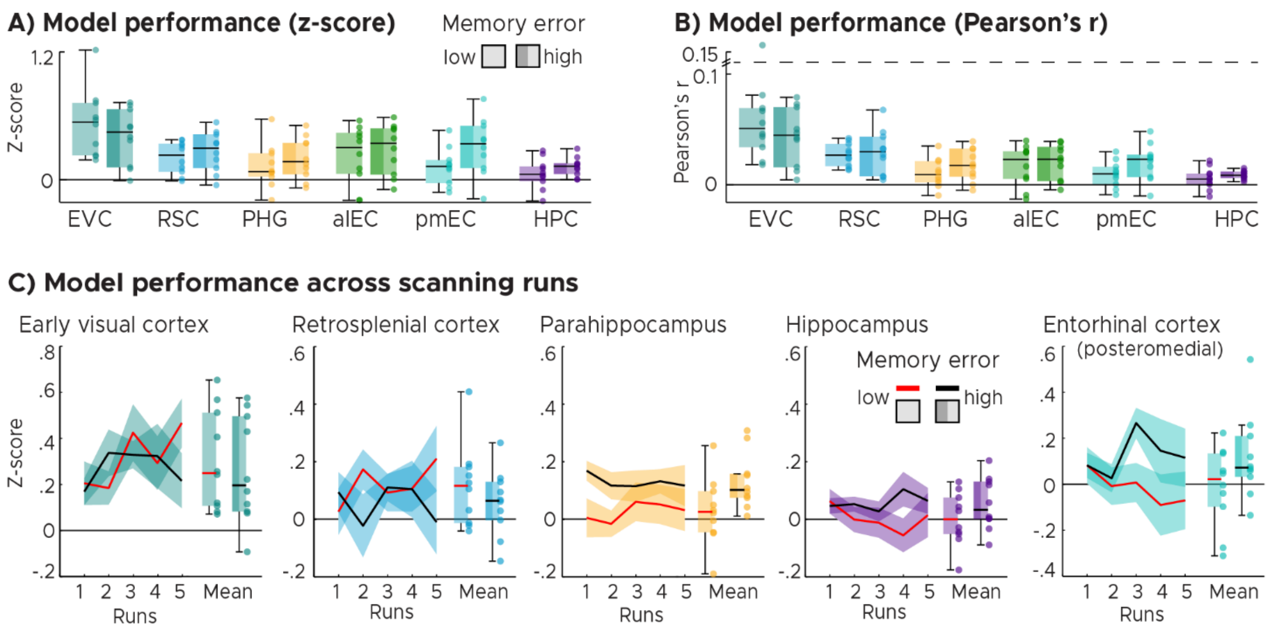

Supplementary figure 8: Optimal model performance expressed in Z-scores (A) and Pearson's R (B) selected for each individual participant instead of group level (Fig. 5B,C) as well as directional tuning for all scanning runs (C). All subfigures (ABC) show single-participant data overlaid on group-level whisker-and-box plots (center, median; box, 25th to 75th percentiles; whiskers, 1.5 × interquartile range) for low and high-memory-error groups (n = 2 x 10 participants). B) Y-axis was cut for visualization (dashed line). C) Model performance (see Fig. 5C) across scanning runs. We performed leave-one-run-out cross-validation for voxels within the ROIs to examine how directional tuning develops over time. In each cross-validation loop a different scanning run was taken as test run, while all others served as training runs. We plot the model performance (Z-score) for each run for high and low memory error participants (median-split by memory error) for each ROI in two formats: 1) mean (solid line) and standard error of the mean (shaded area) for each run, as well as 2) the average model performance across runs as group-level whisker-boxplots. Source data are provided as a Source Data file.

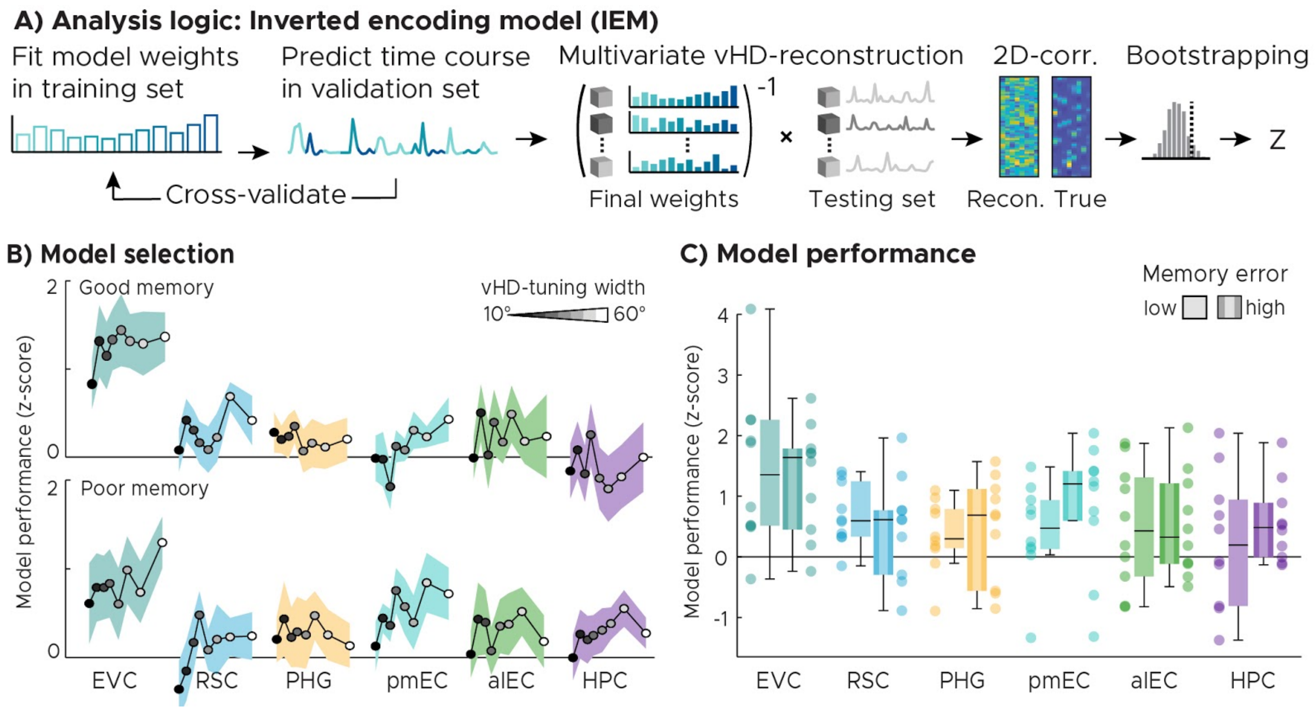

Supplementary figure 9: Multivariate inverted encoding model (IEM). A) Analysis logic. We estimated voxel-wise weights following the same training procedure used for the forward model (see methods). We then multiplied the Moore-Penrose pseudoinverse of all voxel-wise weights in an ROI (m voxels x k weights)-1 with the multivoxel-pattern (m voxels) at each image acquisition (TR) to obtain the estimated vHD-kernel activities at each TR (k weights). We then used 2D-correlation between the reconstructed kernel activities (k weights x n TR's) and the design matrix of the test run (also k weights x n TR's) to compare reconstructed and observed vHD. Finally, the resulting correlation coefficient was converted to a z-score using the bootstrapped null distribution of each ROI (k = 500 unique shuffles). B) Model selection: We plot the IEM-model performance (Z-score) for all basis sets. The black line and the shaded area represent the mean and SEM across participants. Each dot represents the group-average model performance for one basis set, with darker colors representing narrow kernels and lighter colors representing wider kernels. The following kernel widths were tested: 10°, 15°, 20°, 24°, 30°, 36°, 45°, 60°. For each ROI on group-level, we selected the best performing basis set as the optimal model to be tested. C) Optimal model performance selected in B for the two (high- and low-memory-error) participant groups. We plot single participant data and group-level whisker-boxplots (center, median; box, 25th to 75th percentiles; whiskers, 1.5 × interquartile range, n = 2 x 10 participants). Source data are provided as a Source Data file.
