# Supplementary material for: Behavior-dependent directional tuning in the human visual-navigation network
Source: Nat Commun. 2020 Jun 26;11:3247. doi: 10.1038/s41467-020-17000-2 (PMC7320013; doi:10.1038/s41467-020-17000-2)
Supplement: Supplementary file 3 — Reporting Summary [file 41467_2020_17000_MOESM3_ESM.pdf]

nature research

Corresponding author(s): Matthias Nau

Last updated by author(s): 13.05.2020

Reporting Summary

Nature Research wishes to improve the reproducibility of the work that we publish. This form provides structure for consistency and transparency in reporting. For further information on Nature Research policies, see Authors & Referees and the Editorial Policy Checklist .

Statistics

For all statistical analyses, confirm that the following items are present in the figure legend, table legend, main text, or Methods section.

n/a	Confirmed	
☐	☒	The exact sample size (*n*) for each experimental group/condition, given as a discrete number and unit of measurement
☐	☒	A statement on whether measurements were taken from distinct samples or whether the same sample was measured repeatedly
☐	☒	The statistical test(s) used AND whether they are one- or two-sided<br>*Only common tests should be described solely by name; describe more complex techniques in the Methods section.*
☐	☒	A description of all covariates tested
☐	☒	A description of any assumptions or corrections, such as tests of normality and adjustment for multiple comparisons
☐	☒	A full description of the statistical parameters including central tendency (e.g. means) or other basic estimates (e.g. regression coefficient) AND variation (e.g. standard deviation) or associated estimates of uncertainty (e.g. confidence intervals)
☐	☒	For null hypothesis testing, the test statistic (e.g. *F*, *t*, *r*) with confidence intervals, effect sizes, degrees of freedom and *P* value noted<br>*Give P values as exact values whenever suitable.*
☒	☐	For Bayesian analysis, information on the choice of priors and Markov chain Monte Carlo settings
☒	☐	For hierarchical and complex designs, identification of the appropriate level for tests and full reporting of outcomes
☐	☒	Estimates of effect sizes (e.g. Cohen's *d*, Pearson's *r*), indicating how they were calculated

Our web collection on statistics for biologists contains articles on many of the points above.

Software and code

Policy information about availability of computer code

Data collection	We used UnrealEngine2 Runtime software to create a virtual reality arena and to present it inside the MRI machine.
Data analysis	Custom code in Matlab, SPM8 & 12, SnPM, FSL 5.0.4, ANTs.

For manuscripts utilizing custom algorithms or software that are central to the research but not yet described in published literature, software must be made available to editors/reviewers. We strongly encourage code deposition in a community repository (e.g. GitHub). See the Nature Research guidelines for submitting code & software for further information.

Data

Policy information about availability of data

All manuscripts must include a data availability statement. This statement should provide the following information, where applicable:

- Accession codes, unique identifiers, or web links for publicly available datasets
- A list of figures that have associated raw data
- A description of any restrictions on data availability

The source data underlying Figures 1B, 3AB, 4AB, 6B and Supplementary Figures 1A-C, 2C-F, 3ACD, 4A-C, 5AB, 6A-H, 7C-E, 8A-C and 9C are provided as a Source Data file. The virtual navigation data of a sample participant is provided together with analysis code (see Code Availability statement). Other data are available from the authors upon reasonable request.

Field-specific reporting

Please select the one below that is the best fit for your research. If you are not sure, read the appropriate sections before making your selection.

Life sciences study design

All studies must disclose on these points even when the disclosure is negative.

Sample size	Twenty-six participants were recruited as part of an earlier report of our group (Navarro Schröder et al. 2015). No power calculation was performed a priori. The sample size followed the institute's common practices at the time of data acquisition.
Data exclusions	Four participants were excluded because of excessive head motion, i.e. the number of instantaneous movements larger than 0.5mm (Power et al., 2012) exceeded the across-participant average for more than one standard deviation. Another 2 participants were excluded because they finished fewer than four scanning runs. A total of 20 participants entered the analysis.
Replication	Group-level replication was not undertaken but the main effects are present in the majority of participants within each group.
Randomization	Participant group assignment was performed in a data-driven manner based on spatial memory performance
Blinding	The author who analyzed the data did not take part in data acquisition and had no influence on the choice of participants

Reporting for specific materials, systems and methods

We require information from authors about some types of materials, experimental systems and methods used in many studies. Here, indicate whether each material, system or method listed is relevant to your study. If you are not sure if a list item applies to your research, read the appropriate section before selecting a response.

Materials & experimental systems

n/a	Involved in the study	
☒	☐	Antibodies
☒	☐	Eukaryotic cell lines
☒	☐	Palaeontology
☒	☐	Animals and other organisms
☐	☒	Human research participants
☒	☐	Clinical data

Methods

n/a	Involved in the study	
☒	☐	ChIP-seq
☒	☐	Flow cytometry
☐	☒	MRI-based neuroimaging

Human research participants

Policy information about studies involving human research participants

Population characteristics	11 females, 15 males, 19-36 years old
Recruitment	We recruited participants via the local student online forum (Komedia-due) in Duisburg, Germany, via fliers at the University of Duisburg-Essen, Germany as well as at the Donders Institute for Brain Cognition & Behavior (The Netherlands). We are not aware of any selection biases (self or others) that could have impacted the results.
Ethics oversight	The study was approved by the local research ethics committees (ethics committee University Duisburg-Essen, Germany and CMO region Arnhem-Nijmegen, NL) and participants gave written consent prior to scanning

Note that full information on the approval of the study protocol must also be provided in the manuscript.

Magnetic resonance imaging

Experimental design

Design type	Event-related task-based design
Design specifications	Participants navigated in a virtual environment and performed an object-location memory task. They memorized and reported the location of hidden objects inside a virtual arena in the course of 179 trials on average.<br><br>Per participant, we scanned a total of five scanning runs with 210 functional images each. One participant had 220 images per run, and only 4 runs were included for 4 participants due to technical problems during scanning. FMRI repetition time was 2.76 seconds.<br><br>For a part of the fMRI-analyses, participants were split into two groups based on their behavioral performance in the spatial memory task.
Behavioral performance measures	We recorded key-presses corresponding to the participants' navigation behavior (yielding virtual head direction within a 3D-virtual reality (VR)) as well as a measure of spatial memory performance (the Euclidean distance between where the

participants' thought an object was hidden within the VR and where it actually was).

Acquisition

Imaging type(s)

Functional and structural MRI

Field strength

3T

Sequence & imaging parameters

We acquired T2*-weighted functional images on a 7T Siemens MAGNETOM scanner using a 3D-EPI pulse sequence (Poser et al. 2010), a 32-channel head coil and following parameters: TR = 2756 ms, TE = 20 ms, flip angle = 14°, voxel size = 0.9 mm x 0.9 mm, slice thickness = 0.92 mm, slice oversampling = 8.3 %, 96 slices with a 210 mm x 210 mm field of view, phase encoding acceleration factor = 4, 3D acceleration factor = 2. In addition, we acquired T1-weighted structural images (MP2RAGE; voxel size: 0.63 mm isotropic) and B0-field maps (gradient echo; voxel size: 1.8 x 1.8 x 2.2 mm)

Area of acquisition

We centered our field of view on the medial temporal lobe of each participant and scanned frontal, occipital, temporal & medial parietal regions. Superior parietal cortices are missing.

Diffusion MRI ☐ Used ☒ Not used

Preprocessing

Preprocessing software

Data were preprocessed using the automatic analysis library (Cusack et al., 2015), utilizing functions of several analysis packages. For each participant, functional images were realigned and unwarped using SPM8, followed by independent component analysis (ICA) denoising using FIX artifact removal implemented in FSL 5.0.4. To improve signal-to-noise ratio, and with it the ICA-detection of noise components, data were smoothed with a Gaussian full-width-at-half-maximum kernel of 2.5 mm. Images were then non-linearly normalized to a group-average EPI-template using the Advanced Neuroimaging Toolbox (ANTs) (Avants et al., 2011) and high-pass filtered with a 128-s cutoff using FSL. Voxel-wise variance explained by the six realignment parameters (x,y,z, pitch, roll, yaw) as well as by spikes (sudden deviations in signal intensity of more than two temporal standard deviations) was removed via nuisance regression. Out-of-brain voxels were excluded.

Normalization

Images were non-linearly normalized to a group-average EPI-template using the Advanced Neuroimaging Toolbox (ANTs) (Avants et al., 2011).

Normalization template

All analyses were done in a group-average template space specific to these data.

Noise and artifact removal

Functional images were realigned and unwarped using SPM8, followed by independent component analysis (ICA) denoising using FIX artifact removal implemented in FSL 5.0.4. They were high-pass filtered with a 128-s cutoff using FSL. Voxel-wise variance explained by the six realignment parameters (x,y,z, pitch, roll, yaw) as well as by spikes (sudden deviations in signal intensity of more than two temporal standard deviations) was removed via nuisance regression.

Volume censoring

Out of brain voxels were excluded.

Statistical modeling & inference

Model type and settings

Voxel-wise encoding modeling: First, we use voxel-wise L2-regularized regression to obtain weights for each regressor in the design matrix for each voxel. We then use these weights to predict the time course of each respective voxel in independent data. This is iterates across many design matrices. See Figure 2.

Multivariate inverted encoding modeling: We invert the encoding model weights estimated on parts of the data to reconstruct kernel activities from independent data (Supplementary Figure 9).

Effect(s) tested

Key effects: We train a voxel-wise encoding model of virtual head direction (vHD) on parts of the data and use the resulting model weights (one per vHD) to predict the time course of each voxel in independent data using Pearson's correlation. We use weight-shuffling on the testing stage to convert the Pearson's R's to Z-scores. Z-scores were averaged within each Region of interest and tested against zero using permutation-based one-sample t-tests on group level as implemented in the mult_comp_perm_t1 function distributed by Mathworks (https://se.mathworks.com/matlabcentral/fileexchange). In addition, we compare model performance and vHD tuning width across participant groups using permutation-based unpaired two-sample two-tailed t-tests as implemented in statcond distributed via the EEGLab Matlab toolbox (https://github.com/openroc/eeglab/blob/master/tags/EEGLAB7_0_0_0beta). For voxel-wise inference we performed permutation-based one-sample t-test of model performance (Pearson correlations) against zero (k = 10000 shuffles, input image and variance smoothing: 7.2mm) using the Statistical non-parametric mapping toolbox (SnPM)

Specify type of analysis: ☐ Whole brain ☐ ROI-based ☒ Both

Anatomical location(s)

The hippocampal (HPC), anterolateral entorhinal (alEC) and posteromedial entorhinal (pmEC) ROIs were defined manually using ItK-SNAP (www.itksnap.org) based on the high-resolution group average EPI-template. The entorhinal masks were based on previous reports (Navarro Schröder et al., 2015), in which the entorhinal mask was divided into anterolateral (alEC) and posteromedial entorhinal cortex (pmEC). The ROIs for the parahippocampal gyrus (PHG) as well as the retrosplenial cortex (RSC) were based on the reverse inference meta - analysis for “Retrosplenial cortex” and “Parahippocampal cortex” using Neurosynth (Yarkoni et al., 2011). We took the top 5% highest probability voxels from each respective

Neurosynth map and removed isolated voxels from the resulting binary masks. This procedure resulted in coherent bilateral clusters in the medial parietal cortex and parahippocampal gyrus respectively. The early visual cortex (EVC) ROI was created by thresholding the corresponding probability map 'Visual_hOc1' of the SPM anatomy toolbox at 50% and co-registering it non-linearly to our group-average template space.

Statistic type for inference (See Eklund et al. 2016): Voxel-wise

Correction: FDR correction

Models & analysis

n/a	Involved in the study
✗	☐ Functional and/or effective connectivity
✗	☐ Graph analysis
☐	✗ Multivariate modeling or predictive analysis

Multivariate modeling and predictive analysis: Our main analysis estimated the directional tuning of a voxel in several steps. First, we built a virtual head direction (vHD) encoding model by incorporating the participant's navigation behavior into basis sets of circular-gaussian von-Mises distributions, which we call vHD-kernels. Each individual direction was modeled with a different vHD-kernel. Second, we estimated voxel-wise weights for each of these kernels, together representing a voxel's tuning curve. We refer to this step as model training. Third, we used these weights to predict activity in held out data which constituted the model test. This way we obtained a measure of model performance for the given vHD basis set. Finally, by iteratively varying the full-width-at-half-maximum of the vHD-kernels in the basis set and repeating above mentioned steps, we not only tested one vHD-basis set, but multiple ones. This approach allowed us to also estimate the tuning width of each voxel (the kernel width that maximized prediction accuracy).
